# Environmental and socio-demographic individual, family and neighborhood factors associated with children intestinal parasitoses at Iguazú, in the subtropical northern border of Argentina

**DOI:** 10.1371/journal.pntd.0006098

**Published:** 2017-11-20

**Authors:** Maria Romina Rivero, Carlos De Angelo, Pablo Nuñez, Martín Salas, Carlos E. Motta, Alicia Chiaretta, Oscar D. Salomón, Song Liang

**Affiliations:** 1 Instituto Nacional de Medicina Tropical, INMeT. Ministerio de Salud de la Nación. Puerto Iguazú, Misiones. Argentina; 2 Consejo Nacional de Investigaciones Científicas y Técnicas, CONICET. Ciudad Autónoma de Buenos Aires, Buenos Aires, Argentina; 3 Instituto de Biología Subtropical (IBS, UNaM-CONICET). Puerto Iguazú, Misiones. Argentina; 4 Assoc. Civil Centro de Investigaciones del Bosque Atlántico (CeIBA). Puerto Iguazú, Misiones. Argentina; 5 Departamento de Patología Animal, Facultad de Agronomía y Veterinaria, Universidad Nacional de Rio Cuarto. Rio Cuarto, Córdoba. Argentina; 6 Department of Environmental and Global Health, University of Florida, Gainesville, Florida, United States of America; 7 Emerging Pathogens Institute, University of Florida, Gainesville, Florida, United States of America; University of Queensland School of Veterinary Science, AUSTRALIA

## Abstract

**Background:**

Intestinal parasitoses are a major concern for public health, especially in children from middle and low-income populations of tropical and subtropical areas. We examined the presence and co-infection of parasites in humans as well as parasitic environmental contamination in Puerto Iguazú, Argentina. We explored the environmental and socio-demographic characteristics of the persistence of parasites in children and their environment.

**Methodology/Principal findings:**

This cross-section survey was conducted among children population comprised into the area of the public health care centers of Iguazú during June 2013 to May 2016. Copro-parasitological status of 483 asymptomatic children was assessed. Simultaneously, a design-based sampling of 744 soil samples and 530 dog feces was used for characterize the environmental contamination. The 71.5% of these sites were contaminated. Sixteen genera were detected in the environment being hookworms (62.0%) the main pathogens group detected followed by *Toxocara spp* (16.3%), *Trichuris spp* (15.2%) and *Giardia* (6.5%). Total children prevalence raised 58.8%, detecting twelve genera of parasite with *Giardia intestinalis* as the most prevalent pathogen (29.0%) followed by *Enterobius vermicularis* (23.0%), *Hymenolepis nana* (12.4%) and hookworms (4.4%). Through questionnaires and census data, we characterized the socio-demographics conditions at an individual, family and neighborhood levels. A multi-level analysis including environmental contamination data showed that the ´presence of parasites´ was mostly determined by individual (e.g. age, playing habits, previous treatment) and household level (e.g. UBN, WASH, mother's literacy) determinants. Remarkably, to define the level of ‘parasite co-infection’, besides individual and household characteristics, environmental factors at a neighborhood level were important.

**Conclusion/Significance:**

Our work represents the major survey of intestinal parasites in human and environmental samples developed in the region. High prevalence was detected in the children population as well as in their environment. This work shows the importance of considering and promoting multi-level actions over the identified determinants to face this public health problem from integrative approaches.

## Author summary

Enteroparasites are among the main issues in public health arena, especially in children of vulnerable communities from developing countries. We performed a combined analysis of the factors that describe parasite prevalence in children and their environment. Our result evidenced a combined effect of socio-demographic and environmental factors acting at different scales (individual, family, and neighborhood) to determine the parasitic infections patterns and health risk of children at Iguazú Municipality in Argentina. Therefore, this work shed light on the multiple and multilevel factors involved in parasitic diseases, emphasizing the need for action in socio-health and environmental structural issues, not only from the decision-making authorities but also from the individuals, families and communities in favor of promoting healthy environments and healthy individuals.

## Introduction

Infectious diseases associated with the gastrointestinal tract continue to be a major public health concern, especially in middle and low-income populations from tropical and subtropical areas of the developing world [1, 2]. Gastrointestinal parasitoses caused by intestinal protozoan (IP) (e.g. *Giardia intestinalis*, *Cryptosporidium* spp. and *Entamoeba histolytica*) and Soil Transmitted Helminths (STH, referring to *Ascaris lumbricoides*, *Trichuris trichiura*, and hookworms) are endemic and the most prevalent parasitic infections in these regions [3]. Most of these gastrointestinal parasitoses are neglected tropical diseases (NTDs) and they are included in the WHO goals 2020 for the control or elimination of NTDs [3, 4]. Among the reasons for this high prevalence are the multiple factors involved in the maintenance and propagation of these pathogens.

There is agreement about the crucial role of the social and economic context in human health [2, 5], but also about the important role of environmental factors influencing the health of the population [6, 7]. In the last decade, a multilevel theoretical platform for health has been established from an eco-social perspective, which emphasizes a systemic way in understanding determinants of health [8, 9]. All these can be envisioned in the “One Health” paradigm which emphasizes integration among the disciplines from the environmental, human and animal health sectors, endorsing a pluralistic vision on public health issues [8–10].

The social and environmental factors underlying the transmission of intestinal pathogens are varied and different in nature. Enteroparasites infections are commonly associated with the age [11–13], hygiene habits and nutritional or immunological conditions [14–18] of the affected children, indicating the importance of the characteristics at the individual level. Socio-environmental factors at the family or community levels also have been demonstrated as critical because of the relationship between intestinal parasites and WASH inequalities (water, sanitation & hygiene) [19–22], parents’ education level [23, 24] and sanitization and health of pets [25–28] among others. Given the characteristics of the life cycle of intestinal parasites, the environment is also a key player in the maintenance of these infections either as sites of maturation to their infective forms as well as dispersion vehicle [1, 29]. Although there are studies that address these different factors [11, 18, 30, 31], they used to deal with few components or only at one level or scale preventing the possibility of weighing and interpreting all these factors and levels acting together.

Puerto Iguazú is a border municipality located in the Misiones province at the northeastern extreme area of Argentina. Although socioeconomic and physical environments of this region are speculated in favor of high prevalence of intestinal parasites for Misiones province [32], information is very limited and restricted to the central area of the province [18, 33–35]. In Puerto Iguazú there is no systematic research on intestinal parasites despite sporadic information pointing to the possible importance of STHs as a public health issue in the area. The population of Iguazú is among those fastest growing in the nation in the last ten years [36], promoting a rapid transformation of the urban and rural areas [37]. Furthermore, evidence from recent national data suggests that population of this region are among the poorest in the country [38]. In addition, the confluence of immigrants from several European countries, mestizo population, and aboriginal communities *Mbyá-Guaraní*, suggest a great socio-cultural diversity, heterogeneity of socio-economic conditions among the region’s residents and their inequalities in the access to the health system [39]. The epidemiologic importance increases since the municipality borders with Paraguay and Brazil, two countries with a high prevalence of STHs and IP [40–42], and the city represents an important tourist center because of the Iguazú falls, one of the new Natural World Wonders.

Taken into consideration all these aspects, the main goals of this study are to examine the human prevalence of enteroparasites and parasite environmental contamination in Puerto Iguazú and, through a multilevel approach, to explore environmental and socio-demographic characteristics of the persistence of parasites in children and their environment.

## Methods

### Ethics statement

Ethical approval was obtained from Bioethical Committee of the Hospital Dr Ramón Madariaga from Posadas city at Misiones province, Argentina. We especially attend national regulation concerning personal data protection national law No. 25.326/2000 and the principles expressed in the Declaration of Helsinki. Written informed consent from parents or legal guardians was obtained during a household visit that included an explanation of study significance, participant requirements and rights, data about samples collection and socio-demographics questionnaires as well as the opportunity to ask questions. In order to manage illiteracy parents, besides oral explanations, illustrated instructions were also included with the collection kit. Each Public Health Care Center provided anti-parasitic treatment to each positive child under the consultation of a physician. In those cases which demand treatment for the family group, it was properly provided. The national drug policy throughout the REMEDIAR program (http://www.remediar.msal.gov.ar/) manages and ensures drugs distribution and availability to each PHCC. No follow-up stool samples analysis after treatment was performed since it was beyond the scope of the project. It was not needed the Institutional Review Board approval for the collection and use of dogs´ feces samples since they were collected from public places and the individual characterization was not performed.

### Study area

**Fig 1:**
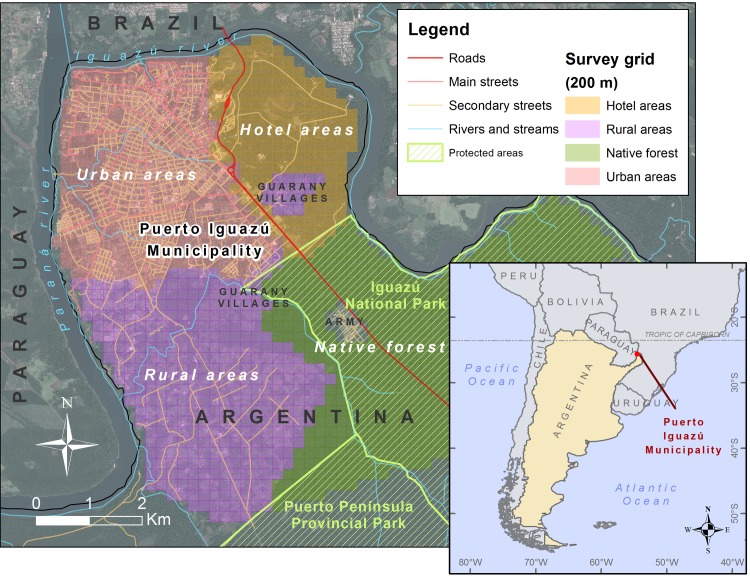
Study area. Location of the Iguazú Municipality in the tri-border area of Argentina, Brazil and Paraguay (inset) and detail of the main areas of the study area showing the grid used for the environmental survey. This map was created with ArcGIS 10.4 (www.arcgis.com).

Puerto Iguazú city is located in Misiones province (25°35‘52”S and 54° 34’ 55”W), a subtropical province of northeastern Argentina (Fig 1) and it is part of the most biodiverse region in Argentina (named the Upper Parana Atlantic Forest ecoregion) [39]. The region is characterized by a subtropical climate with no dry season. The predominant soil type is lateritic of deep red color [39]. The municipality includes the city of Puerto Iguazú, peri-urban and rural areas, and a large area covered by native forests (parks and reserves). There are also *Mbyá Guaraní* aboriginal villages at city periphery.

### Study design

Cross-sectional surveys on humans and environment were carried out during June 2013 and May 2016 in Puerto Iguazú city. It involved two main simultaneous study designs (see below) directed to assess the environmental contamination of intestinal parasites and entero-parasitic infections among children. Public areas were included for the environmental survey and for the children survey we assessed the neighborhoods around the Public Health Care Centers (PHCC) of the Municipality. All the areas of the Municipality were sampled simultaneously trying to maintain a uniform sampling effort along the study. Child population of aboriginal villages was not included in this study since they were involved in a specific program of aboriginal health. All samples were delivered to and processed at the laboratory of Parasitology and Zoonotic Diseases of the National Institute of Tropical Medicine (PZD-INMeT) of Puerto Iguazú. As the aim of this study was a general characterization of the occurrence and co-occurrence of parasites in the environment and in the children of Iguazú, samples were classified as positive if there was an egg or oo/cyst observed in one or more of any slide in any technique applied for its diagnosis. The analyses were conducted in duplicate by two experienced microscopists. As quality control, a random 10% of the total samples were re-examined by a senior laboratory researcher. Socio-environmental covariates were obtained from the analysis of local and landscape variables as well as socio-demographic data obtained from the last national census in 2010 and collected from questionnaires performed in the first visit to a household participating in the study (see more details below).

### Assessing environmental contamination

#### Environmental sampling

The survey included a collection of soil and dogs’ feces samples and subsequent parasitological examination. To ensure a representative and sufficient geographical coverage, a design-based sampling was performed using a regular grid of 200x200-m sub-areas overlaid on the Iguazú municipality map using GIS (Fig 1). From the complete grid, total of 232 cells were randomly selected and a square, a street or a vacant lot into each cell was designated as a sampling site in each cell. In the situations where the conditions for access to the cells were difficult, a neighboring cell was selected.

Four samples of soil from 4 different locations were collected in a 20-m radius from each sampling site, making a total of 744 samples. At each location, approximately 300 g of top soil or sand was taken using a small shovel in the area inside a square 15×15 cm and 5 cm in depth. The samples were taken from the sites without grass and were stored in labeled plastic bags. Up to five dogs’ feces detected around each sampling site were collected as a pool in a single labeled recipient containing formalin 10% totalizing 530 stool samples from domestic dogs analyzed. Only fresh feces were collected, and all samples were stored at 4°C, and transported to the laboratory at PZD-INMeT.

The geographical coordinates of each sampling site were determined using a handheld global positioning system device. Local conditions of the environmental sampling site (Table 1) were described at the moment of sample collection.

**Table 1:** Socio-environmental variables.

Level	Group of variables	Variables	Description
***Environmental conditions***	*Landscape scale–topography*	Elevation Distance to rivers Slope Orientation	S1 and S2 Tables
	*Landscape scale—social and economic conditions*	Street density Population density Inadequate services Overcrowding Unsatisfied Basic Needs	
	*Landscape scale—land cover*	Trees Grass Bare soil Construction Surface temperature	
	*Local scale*	Presence of dogs Presence of farm animals Presence of trash Substrate Latrine	
***Individual***	*Child*	Sex Age group	S3 Table
	*Nutritional status*	Wasted Stunted Underweight Obese or overweight	
	*Exposure*	Previous drug supply	
	*Habits*	Hand washing Wearing shoes Playing with soil	
***Household***	*House*	Economic status Unsatisfied Basic Needs	S4 Table
	*Yard*	Peridomiciliary hygiene Presence of farm animals	
	*Diet habits*	Origin of vegetables	
	*WASH*	Tap water consumption Safe waste disposal	
	*Family*	Children per family Family with more than 3 children Overcrowding Young mother Single mother Working mother	
	*Pests*	Rodents	
	*Environmental risk*	Co-contamination (household area)	
***PHCC area***	*Socio-economic*	Water supply(PHCC area) Water service (PHCC area) Population density (PHCC area) Unsatisfied Basic Needs (PHCC area)	S5 Table
	*Environmental risk*	Co-contamination (PHCC area)	

List and characteristics of socio-environmental variables included in the study. Variables at the environmental conditions’ level were used to characterize the environmental sampling sites. Variables at the individual, household and Primary Health Care Center (PHCC) levels were used to characterize human samples.

#### Examination of environmental samples

Dogs’ feces were completely processed and examined similarly to the protocol for examination of human stools samples (see below). Accordingly, macroscopic and microscopic examinations were developed.

To obtain the final volume of soil samples, the quartering technique was applied [43]. Soil samples were analyzed by a decantation and centrifugation technique with sequential filtration passages through mesh sieves of micrometer pores, which was based on the modified version [34] of Shurtleff & Averre [44] method and we added slight variations in this work. Briefly, each sample was eluted in 925 ml H20, shacked 15 min, and lend to decantation for 30 min. The supernatant was passed through a mesh sieve of 150 μ pores to remove bigger grass. 250 ml of the cleared solution was then passed subsequently through mesh sieves of 50μ (to retain bigger eggs) and 25μ pores (to retain medium and small eggs) for three times. The residues that remained on the top of each mesh sieves were washed with distilled water and collected in individual tubes. After a final centrifugation to concentrate parasite ova, samples were taken for microscopic inspection. Finally, the liquid volume that passed through both mesh sieves was processed under modified Telemann concentration technique and Kinyoun stain to cyst and oocyst survey. Final characterization of each sampling site was defined by the combined result of all these techniques.

### Assessing parasite infections among children

#### Human sample collection

**Fig 2:**
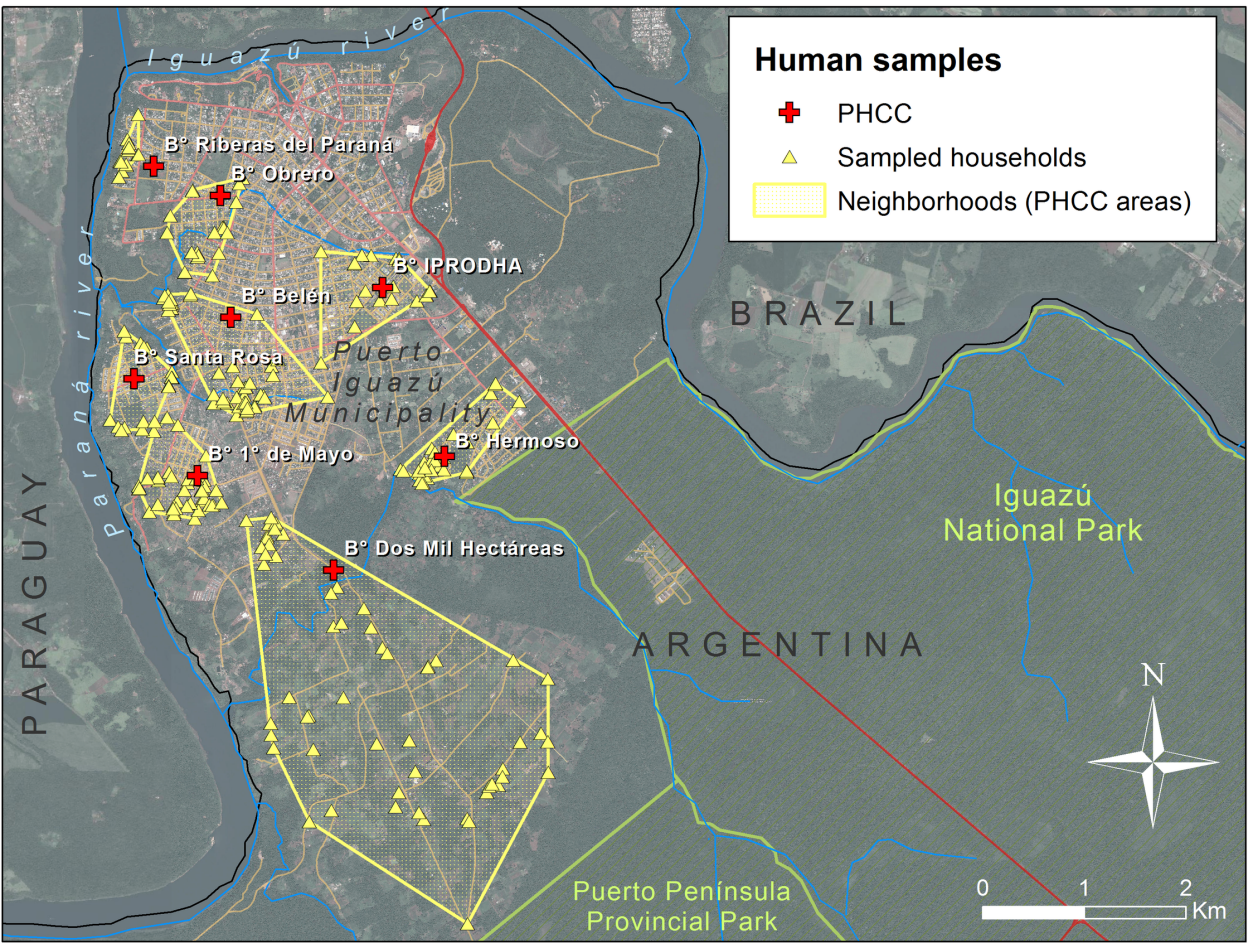
Location of the household surveyed in this study along the Puerto Iguazú Municipality. All the households are grouped by the corresponding Public Health Care Center (PHCC), delimitating the neighborhood by the minimum convex polygon comprising the entire households related to each PHCC. This map was created with ArcGIS 10.4 (www.arcgis.com).

Human sampling was randomized and stratified to nine health care centers areas (PHCC, Fig 2). These PHCC areas are located in urban-periurban and rural areas of the municipality and a file of each child that is assisted by the PHCC is available at the health department. Overall, from these population records, we were able to estimate that about 4800 children <15 years of age were in the PHCC study areas. We attempted to incorporate in this study 10% of the children from each PHCC. A simple random sampling method was used to select the households and children participating in the study. Healthy children without evident parasitic or diarrheal disease (neither under actual treatment nor in the last six months) were incorporated. The recruitment process involved 483 children from 272 families with a mean of 1.80 ± 0.91 children per family.

Families choose their preferred PHCC for closeness, comfort and/or medical benefits it provides. For this reason, to assess the real area covered by each PHCC in this study, we performed a minimum convex polygon based on the data of each participant family obtained in the field, in agreement with the records of each care center (Fig 2). As these polygons containing all the households related to each PHCC did not overlap, we assumed that they provide a proper description of the effective area or neighborhood associated with the PHCCs.

Families’ recruitment and human sample collection occurred simultaneously along different PHCC trying to obtain samples homogeneously distributed along the study. During the first household visit, a socio-demographic questionnaire was addressed to the mothers or tutors (see more details below) and a disposable collection kit was provided to the guardian for each child. They were instructed to follow a serial collection method every other day during a week in order to collect three samples in total. The kit consisted of two plastic containers with SAF (Sodium acetate-acetic acid-formalin) as a preservative solution, spoon, tray, gauzes and gloves. One plastic container was for the stools and the other one was for placing the gauzes used in anal swabs (specific technique for the diagnosis of *Enterobius vermicularis*). Besides oral explanations, illustrated instructions were also included with the kit. All these materials were labeled with the child name and an identification code. Field staffs were trained in proper hygienic and bio-safety measures.

#### Human diagnosis

Coproparasitological status of each child was assessed by complete examination of stool and gauzes. The combined implementation of a serial collection method with different diagnosis techniques optimizes the detection of a diverse parasite spectrum; therefore, parasite infection for each child was determined by the pooled result of all the techniques performed. Firstly, an exhaustive macroscopic examination under a stereoscopic magnifier and direct microscopic examination (direct smear) with Lugol solution were accomplished. Then, each stool sample was subject to Sheather’s flotation and modified Telemann´s techniques [45]. To remove large fecal debris, sieving was performed prior to coprological techniques. Gauzes were subject to centrifugation at 1500 rpm, 10 min and the pellet was observed under light microscopy. Stool examinations were performed over the entire area under the coverslip using a PrimoStar Zeiss optical microscope with dry 10X and 40X objectives. Finally, the Kinyoun [46] and trichromic staining [47] were performed to each sample and observed with wet 100X objective. Further details on these methods for helminth and protozoa diagnosis were presented elsewhere [48–51]. Identification of parasites was performed through morphological and morphometric characteristics.

Stool analysis results were informed by triplicate: parents/guardians of the subjects received oral and written notification of the results, a copy was attached to each child PHCC file and another copy was to the INMeT research files.

### Multi-level analysis

#### Characterization of environmental sampling sites

We characterized the sampling site by its local and landscape conditions (Table 1). For landscape conditions we used a GIS database with the cadastral and census information of the Iguazú city and supervised classifications of satellite images (S1 Table). We generated three groups of variables describing the environment at a regional scale: 1) Topography: terrain elevation, terrain slope, terrain orientation (sun exposure), distance to water courses; 2) Social and economic conditions of the area: density of streets, population density, construction quality, availability of public services, unsatisfied basic needs, among others; 3) Land cover: vegetation (trees and grass), bare soil, construction, and surface temperature (S1 Table). Although these landscape characteristics can be considered approximately stationary at the spatial and temporal scale of the analysis, socio-economic conditions and land-cover layers were created from the available data (national census and satellite images) that were most close to the midpoint of our environmental sampling time frame (see details in S1 Table). At a local scale, we recorded local conditions of hygiene and domestic animal presence in the each study site at the moment of sampling collection (S2 Table).

#### Environmental contamination and its associated factors

To explore the main determinants of environmental contamination by varying enteric parasites, the presence and co-contamination level of parasites at each sample site were used as response variables. The ‘presence of parasite contamination’ was determined by the presence of at least one parasite form such as larvae, eggs or cysts, either in the soil or in the fecal samples collected at each site along the municipality. The ‘co-contamination level’, was determined by the number of different species of parasites detected in each sampling site combining soil and dog samples.

The environmental characteristics of the sampling sites described above were included in the analysis as explanatory variables (fixed effects) in generalized linear models (GLM) developed for both response variables. The presence of contamination was modeled as a binary response variable with a logit-link function, and the co-contamination (count data) was modeled using a Poisson error distribution [52]. Firstly, univariate analyses were performed to select important variables (e.g. statistically significant covariates)[53], and in a second step the most important intependent variables were analyzed by groups (see groups in Table 1) in subsequent multivariate GLM analyses. Before combining variables in multivariate models, we checked for multicolinearity inferred by variance inflation factor >5 [52]. When two or more correlated variables were detected for candidate models, only the most significant variables in the univariate models were included in further models.

The models obtained for each group of variables and their possible combinations were compared hierarchically using the Akaike Information Criterion corrected for small samples (AICc) to identify the best fit models containing only the most important and uncorrelated variables of each group [54]. General goodness of fit of the models was assessed by the AICc comparison and (log)-likelihood ratio tests, while overdispersion was assessed by the scale parameter to ensure that no further corrections were necessary [52, 55]. Final models were verified and evaluated estimating the area under the receiver operating characteristic curve (AUC, for binomial models) and the Spearman rank correlation between observed and predicted levels of co-contamination (for Poisson distributed models) [56, 57]. As with the final models with landscape variables we attempted to predict parasite contamination in new areas (see below), for these specific models the validation parameters were estimated through a 2-fold cross validation [53, 56, 57]. For this procedure, we randomly divided the original data in two subsets, and we used one of these subsets for building the model (the training subset) and the other one for model validation (the validation subset). Then, we inverted the role of the training and validation subsets, and we reported the mean prediction capability from the two subsets for each model (mean cvAUC or mean cvSpearman Rank rho with their p values). Statistical analysis and model selection were carried out with R [58] with packages *car* [59] and *MuMIn* [60]. The package *cvAUC* [61] was used for model evaluation. Potential spatial autocorrelation was assesed in the residuals of the final spatial models using the Moran's I index [62] in ArcGIS 10.4.

#### Environmental risk maps

To develop risk maps representing the levels of parasite contamination in the environment, only the models that showed significant predictive capacity were used. If the final environmental GLM contained both landscape and local variables, only the landscape model was selected for constructing the contamination risk map because the local condition variables were not explicit geographically in unsurveyed areas. We extrapolated the final model or models to the whole study area using GIS and the corresponding link function for the GLM (logit link for binomial models or log-link for Poisson models). To spatially represent the uncertainty of this map or maps, we also extrapolated the model 95% confidence interval to the study area.

To estimate the environmental contamination level associated with each household that participated in the human sample study, we overlaid this risk map or maps with the distribution of the households (‘Environmental risk at the household level’, Table 1). To estimate the mean environmental contamination level for each neighborhood or PHCC area, we used the minimum convex polygon estimated for each PHCC (Fig 2) and we estimated the average contamination level for the PHCC area according to our risk maps (‘Environmental risk at the PHCC area’, Table 1). GIS management for variable development and risk map construction was made using ArcGIS 10.4 and Spatial Analyst (ESRI Inc.).

#### Socio-demographic conditions: Individual and household levels

Socio-demographic data were acquired by implementing a pre-tested and structured questionnaire administered to children’s mothers during house-to-house visits. The questionnaires were organized with fixed questions (the dichotomous type and multiple choices); the terminology used was brief, respectful, accurate, objective and everyday language, to facilitate a quick response. All recorded parasitological and questionnaire data were double-entered into a database and cross-checked.

The questionnaire allowed the survey of more than 100 socio-demographic variables which in turn gave rise to new categorical variables product of the grouped analysis, including habits, hygiene, demographic, family and household related variables (Table 1, S3 and S4 Tables).

Additionally, we evaluated the nutritional status to assess the partial effect on parasite and co-infection prevalence. Individual data for height (cm) and weight (kg) were collected at the corresponding PHCC for anthropometric analyses. Z-scores were calculated for weight for age (WAZ), weight for length/height (WHZ), length/height for age (HAZ) and body mass for age (BMIZ) using the sex-specific WHO Child Growth Standards through WHO Anthro and WHO Anthro Plus software [63, 64] (see details in S3 Table).

#### Socio-demographic conditions: PHCC level

We characterized the socioeconomic conditions at the PHCC level by calculating the mean population density and the percentage of households with Unsatisfied Basic Needs (UBN) in the area according to the minimum convex polygons for each center as explained above and data from the last national census [10].

#### Multi-level socio-demographic and environmental model

Intestinal parasite presence/absence (binary distribution) and multi-parasitism (i.e. co-infection; Poisson distribution) were the response variables in multi-level modeling analyses. We applied generalized linear mixed-effects models (GLMM) that included households and PHCC as random effects to consider the dependence of the data and nested factors (child<household<PHCC) [52]. The number of children per family was considered as a weight argument in these models to balance the potential bias in household level factors caused by families with a high number of children [52]. We assessed the potential effects of the socio-demographic conditions and the environmental contamination as fixed effects. The general modeling and model selection procedure and verification were the same described above for the environmental models using the hierarchical structure of the variables described in Table 1. Statistical analyses were carried out with R software [58]through library *lme4* [65]. Best-fitting models were selected on the basis of the Akaike information criterion corrected for small samples (AICc) through library *MuMIn* [60].

## Results

### Characteristics of the study population and households

The child population studied was 54.9% male, with a mean age ± SD of 5.8 ± 3.5 years old. Underweight affects 12.8% of the children, while the stunting involves 11.3% and wasting 12.7% of the sample. Overweight and obesity together affected 18.1% of the studied children. The mean age of the mothers was 30.8 ± 8.2 years old and 19.5% were single mothers. The employment situation of the families was precarious and most of the mothers (83.2%) were homemakers. The 26.7% of the children has more than three siblings with the presence of overcrowding in 53% of the households. Sixty-three percent of the parents possess primary-level education only, and most of the children (88.6%) reside in UBN households with no peri domiciliary hygiene (65.4%). Drinking water, excreta disposal and waste disposal were not safe in around 40% of the households (44.3, 41.2 and 43.5% respectively). Most of the houses (75.8%) were constructed with none or just one main component of cement, being land and wood other predominant materials. The 21.5% of the households possesses farm animals and almost 80% of the families have dogs (1.8 ± 1.8 dogs per family).

### The parasites in the tri-border city of Iguazú

#### Environmental contamination (soil and dog feces)

**Fig 3:**
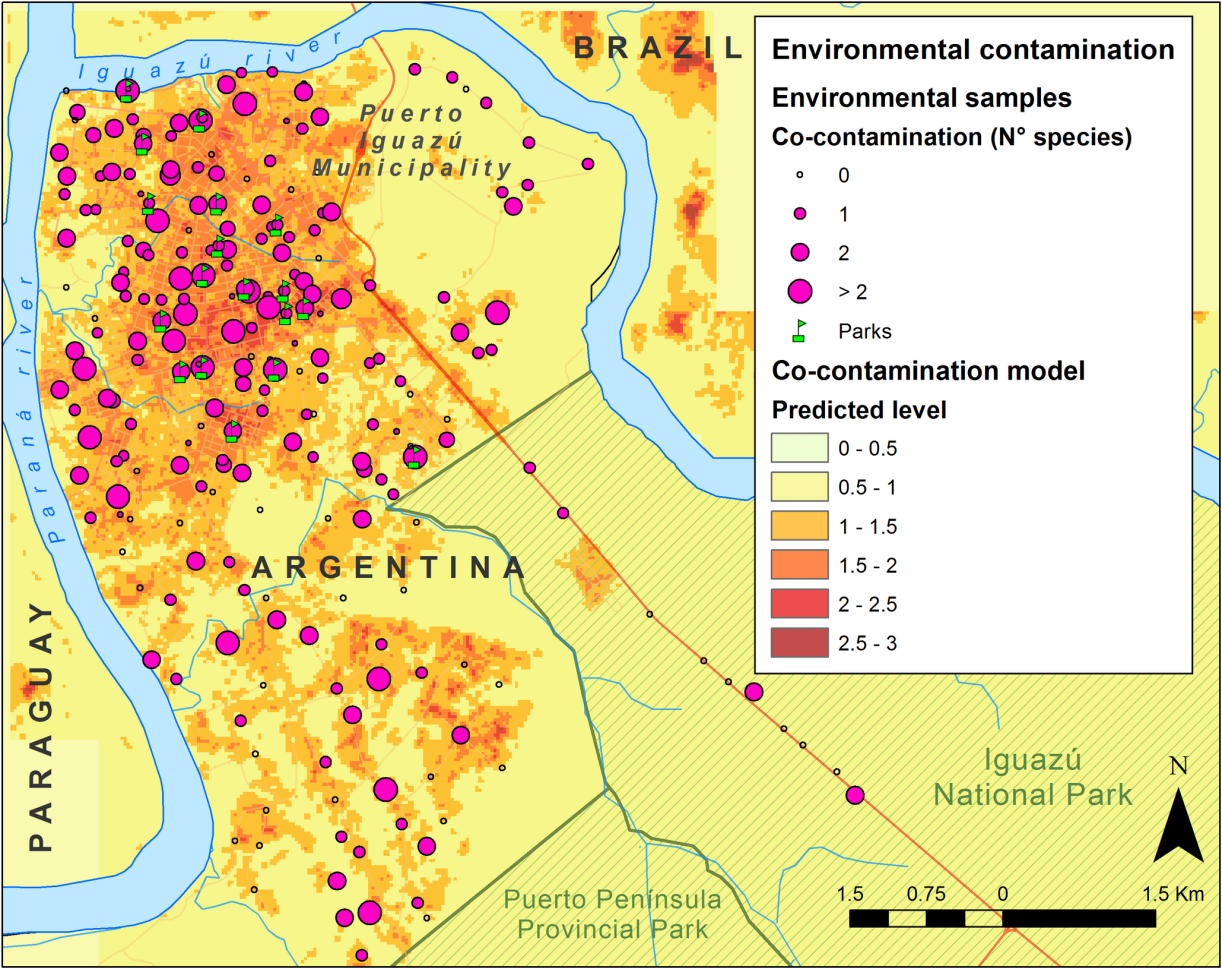
Predicted co-contamination with parasites in the Iguazú Municipality. Survey results and map of the predicted of number of parasite species (co-contamination). The parks of the city under study are shown in the figure. This map was created with ArcGIS 10.4 (www.arcgis.com).

In total, 166 (71.5%) out of 232 surveyed sites were positive for at least one helminth or intestinal protozoan species (Fig 3). Most of the dog fecal samples (63.8%) and 37.5% of soil samples contained parasites (Table 2). The soil contamination seemed to correlate well with positive dog fecal samples collected at the same site (χ2 = 3.727, df = 1, p = 0.053).

**Table 2:** Environmental contamination with parasite structures and parasite infection in asymptomatic children.

Parasite species or groups	Prevalence (%)			Co-contamination (%)					Prevalence (%)	Multi-parasitism in children (%)			
	Dog	Soil	Site	1 sp	2 spp	3 spp	4 spp	5 spp	Children	Mono	Double	Triple	Quadruple
*General*	63.8	37.5	71.5	45.1	38.3	11.3	4.5	0.8	58.8	65.8	28.2	5.3	0.7
**Helminths**													
*Ascaris* spp.	0.9	1.1	2.2	0.0	25.0	0.0	50.0	25.0	0.6	0.0	66.7	33.3	0.0
*Capillaria* sp.	0.0	1.1	1.1	0.0	100.0	0.0	0.0	0.0	-	-	-	-	-
*Diphyllobothrium* spp.	0.9	0.0	1.1	0.0	50.0	50.0	0.0	0.0	-	-	-	-	-
*Dipylidium caninum*	1.3	1.6	3.3	16.7	33.3	33.3	16.7	0.0	-	-	-	-	-
*Enterobius vermicularis*	-	-	-	-	-	-	-	-	23.0	56.3	37.5	5.4	0.9
Hookworms	55.6	28.8	62.0	41.2	39.5	13.2	5.3	0.9	4.4	38.1	42.9	19.0	0.0
*Hymenolepis nana*	0.0	2.2	2.2	25.0	25.0	25.0	25.0	0.0	12.4	21.7	56.7	18.3	3.3
*Strongyloides stercoralis*	-	-	-	-	-	-	-	-	1.9	22.2	11.1	55.6	11.1
*Taenia* spp.	0.4	0.0	0.5	0.0	0.0	100.0	0.0	0.0	-	-	-	-	-
*Toxascaris leonine*	3.9	1.6	6.0	9.1	90.9	0.0	0.0	0.0	-	-	-	-	-
*Toxocara* spp.	13.4	6.0	16.3	13.3	43.3	33.3	10.0	0.0	-	-	-	-	-
*Trichuris* spp.	12.1	3.8	15.2	7.1	53.6	21.4	14.3	3.6	-	-	-	-	-
**Protozoans**													
*Blastocystis hominis*	0.4	0.0	0.5	0.0	100.0	0.0	0.0	0.0	2.1	70.0	20.0	10.0	0.0
*Cryptosporidium* sp.	-	-	-	-	-	-	-	-	0.6	100.0	0.0	0.0	0.0
*Eimeria* sp.	0.9	0.0	1.1	50.0	50.0	0.0	0.0	0.0	-	-	-	-	-
*Endolimax nana*	-	-	-	-	-	-	-	-	0.8	25.0	50.0	25.0	0.0
*Entamoeba coli*	0.9	0.0	1.1	0.0	50.0	50.0	0.0	0.0	6.4	51.6	32.3	12.9	3.2
*Entamoeba histolytica*	-	-	-	-	-	-	-	-	1.0	40.0	60.0	0.0	0.0
*Giardia intestinalis*	6.9	0.0	6.5	0.0	33.3	25.0	33.3	8.3	29.0	57.9	35.0	5.7	1.4
*Iodamoeba bütschlii*	0.0	0.5	0.5	0.0	0.0	0.0	0.0	100.0	0.4	0.0	50.0	0.0	50.0
*Isospora canis*	1.7	1.6	3.8	28.6	28.6	28.6	14.3	0.0	-	-	-	-	-

The prevalence shows the proportion of contaminated soil samples and sampling sites, and the percentage of infected dog and human samples. The co-contamination and multi-parasitism in children show the percentage of sites or children with the different levels of parasitism, and the frequency distribution of each species or species’ group along the different levels.

At least 16 genera of parasites were identified, with the majority having zoonotic potential (Table 2). Hookworm was the most prevalent parasite in both soil and dog fecal samples, accounting for 28.8% and 55.6%, respectively; followed by *Toxocara canis* and *Trichuris vulpis*. Also, lower prevalence of *A*. *lumbricoides* was found (Table 2). *Giardia intestinalis* was the most prevalent protozoan and it was only detected in dog samples. *Isospora canis* was the only protozoan parasite detected in both, soil and dog samples, but it was presented in a few cases (Table 2). Two dog samples were positive for *Diphyllobothrium* spp eggs.

Most of the sites presented low co-contamination, with one or two species of parasites per site, and 16.5% of the sites presented higher intensities with more than two species (Table 2; Fig 3). In dogs, co-infections were detected in 30.4% of the fecal samples. All samples collected in parks with children playground (Fig 3) were positive for parasite contamination and the most prevalent parasitic eggs belonged to *Ancylostomidae* family.

#### Parasite identification and prevalence in children

The overall prevalence of infections among children by one or more intestinal parasites was 58.8% (284/483). From the positive cases, a total of 65.85% corresponded to mono-parasitism, whereas 34.15% corresponded to multiple-parasitism cases. *E*. *vermicularis* and *G*. *intestinalis* were the most common mono-infections. The co-infection with these two parasites was the most frequent form of biparasitism followed by *H*. *nana* and *G*. *intestinalis*. In addition, we found that co-infections among children could be up to four different genera (Table 2). Children between 5 to 9 years old showed a higher prevalence of any infection (70%) and multi-parasitism (48%), whereas children under 5 years old presented a lower prevalence of parasites (51%) and multi-parasitism (12.4%). Prevalence of infections was similar between boys (60%) and girls (58%). Similar prevalence of parasites was observed for stunted and not stunted children (60% and 58%, respectively), as well as for wasted and underweight categories. However, the prevalence of parasites was higher in not obese or overweight children (60%) compared to obese or overweight (48%).

### Environmental risk of parasite contamination

Environmental contamination largely occurred in the most urbanized areas and increased where trash was present in the area. In the univariate analysis (S6 Table), the presence and co-contamination of parasites (i.e. the number of species found) was higher in the areas in lower elevations, a higher density of streets, lower cover of trees, and higher surface temperature. At a local scale, both environmental contamination and co-contamination were positively associated with the presence of trash in the streets and negatively related to the presence of latrines in the area of sample collection (S6 Table). Combining these results in a multivariate analysis (S7 and S8 Tables) suggested that the presence of trash (local variable) and the street density (landscape variable) were the most important determinants for the presence of parasite contamination (Table 3, models A1 –A3). For co-contamination estimates, the presence of trash and the surface temperature (landscape variable) were the most important predictors (Table 3, models B1 –B3).

**Table 3:** Factors predicting environmental contamination with parasites structures.

Presence of parasite contamination				Co-contamination with parasite structures			
Fixed effects	Estimate	Std. Error	p	Fixed effects	Estimate	Std. Error	p
**A1. Landscape model for presence of contamination** *G*^2^ = 11.514, df = 1, p<0.001; cvAUC = 0.667				**B1. Landscape model for co-contamination** *G*^2^ = 18.387, df = 1, p<0.001; mean rho = 0.316 ± 0.02, p **<**0.001			
(Intercept)	0.193	0.272	0.479	(Intercept)	-42.629	10.274	**<0.001**
***Landscape scale–socio-economic***				***Landscape scale—land cover***			
Street density	0.019	0.006	**0.001**	Surface temperature	0.144	0.034	**<0.001**
**A2. Local model for presence of contamination** *G*^2^ = 5.915, df = 1, p = 0.015; AUC = 0.602				**B2. Local model for co-contamination** *G*^2^ = 4.616, df = 1, p = 0.032; rho = 0.187, p **=** 0.021			
(Intercept)	0.288	0.289	0.319	(Intercept)	-0.063	0.147	0.663
***Local scale***				***Local scale***			
Trash	0.904	0.371	**0.015**	Trash	0.356	0.170	**0.037**
**A3. Combined model for presence of contamination** *G*^2^ = 11.446, df = 2, p = 0.003; AUC = 0.653				**B3. Combined model for co-contamination** *G*^2^ = 15.147, df = 2, p<0.001;; rho = 0.333, p **<**0.001			
(Intercept)	-0.371	0.409	0.364	(Intercept)	-41.523	12.916	**0.001**
***Landscape scale–socio-economic***				***Landscape scale—land cover***			
Street density	0.015	0.007	**0.023**	Surface temperature	0.139	0.043	**0.001**
***Local scale***				***Local scale***			
Trash	0.961	0.381	**0.012**	Trash	0.373	0.170	**0.029**

Final models obtained for predicting parasite contamination (A) and co-contamination (B) in the environment at the Iguazú Municipality. The goodness of fit (GOF) of the models was evaluated in a hierarchical comparison with other candidate models by the AICc (see S7 and S8 Tables) and (log)-likelihood ratio tests. Binomial models (A1 to A3) were evaluated by the area under the receiver operating characteristic curve (AUC) and the co-contamination models (B1 to B3) by the Spearman’s rank correlation (rho) between observed and predicted levels of co-contamination. A 2-fold cross-validation was used for landscape models.

The model explaining the presence of contamination with landscape variables showed moderated to low prediction capabilities (mean cvAUC = 0.667, 95% CI 0.579–0.753) and therefore it was not used for predicting contamination in the study area. The selected co-contamination model with landscape variables (Table 3, B1) showed moderated but significant prediction capacity in cross validation (mean cvSpearman Rank rho = 0.316 ± 0.02, all p values <0.001), and it did not show signals of spatial autocorrelation in its residuals (Moran's Index: 0.009, z-score: 0.302, p = 0.763). Therefore, this model was used for characterizing the contamination degree of the study area (Fig 3). The predicted co-contamination showed a heterogeneous spatial distribution along the Iguazú Municipality, but with higher values in the central areas of the city due to the association of elevated surface temperatures with the most urbanized areas, where higher co-contamination levels were also explicit in the predictions of the lower and upper limits of the 95% confidence interval (S1 Fig).

### Multi-level determinants of parasite diseases

Parasite infection and co-infection of Iguazú children were determined by several factors that affect them at different levels (Table 4). At the individual level, the age was one of the most important factors in children between 5 and 9 years old showing higher probabilities of infection and co-infection. Playing with soil is a strong predictor of both infection and co-infection and previous anti-parasitic treatment was found as a significant risk factor for infection. Regarding nutritional status, there was no a clear association between childhood undernutrition (stunting, wasting and underweight) and parasites presence, although obese and overweight children showed lower probabilities of being infected.

**Table 4:** Multilevel factors affecting children infection and co-infection with parasites.

Fixed effects	Estimate	Std. Error	p	Fixed effects	Estimate	Std. Error	p
**C. Parasite infection in children** *G*^2^ = 81.437, df = 10, p<0.001; AUC = 0.964				**D. Parasite co-infection level in children** *G*^2^ = 46.991, df = 6, p<0.001; Spearman Rank rho = 0.343, p < 0.001			
(Intercept)	-35,282	12,021	**0.003**	(Intercept)	-13,156	0.3409	**<0.001**
***Individual level***				***Individual level***			
*Age*				*Age*			
Age group 2	0.747	0.263	**0.004**	Age group 2	0.460	0.113	**<0.001**
Age group 3	0.202	0.292	0.489	Age group 3	0.222	0.146	0.129
*Nutritional conditions*				*Nutritional conditions*			
Obese or overweight	-11,952	0.360	**0.001**	-			
*Exposure*				*Exposure*			
Previous deworming treatment	0.581	0.340	0.088	-			
*Habits*				*Habits*			
Playing with soil	20,535	0.484	**<0.001**	Playing with soil	0.201	0.110	0.067
***Household level***				***Household level***			
*House conditions*				*House conditions*			
Unsatisfied Basic Needs	17,137	0.8729	0.050	-			
*WASH*				*WASH*			
Tap water	10,442	0.575	0.069	Safe excreta disposal	-0.322	0.102	**0.002**
*Family*				*Family*			
Single mother	12,028	0.675	0.075	More than 3 child	0.382	0.106	**<0.001**
Mother literacy	-11,029	0.560	**0.049**				
Overcrowding	0.368	0.1855	**0.047**				
				***PHCC level***			
				*Environmental risk*			
				Mean predicted co-contamination	0.630	0.238	**0.008**

Final models obtained for predicting parasite infection (model C: binomial distribution) and co-infection level (model D: Poisson distribution) in the child population of Iguazú Municipality. These models resulted from a GLMM with the household (family) and the Primary Health Care Center (neighborhood) as random effects. The goodness of fit (GOF) of the models was evaluated in a hierarchical comparison with other candidate models by the AICc (see S10 and S11 Tables) and (log)-likelihood ratio tests. Binomial models (C) were also evaluated by the area under the receiver operating characteristic curve (AUC) and the co-infection models (D) by the Spearman’s rank correlation (rho) between observed and predicted levels of co-infection.

At a household level (Table 4), WASH and family variables were important to predict parasite infection and co-infection level, while the household characteristics (e.g. UBN) were significant only for predicting infection. Among the WASH determinants, the safe excretes disposal reduces the level of co-infection, but unexpectedly, the access to tap water increases the probability of infection.

Family composition was also important for both response variables (Table 4). Families with single mothers have higher infection probabilities and larger families (with more than 3 children) showed higher co-infection intensities. Similarly, overcrowding was an important predictor of parasite infection, and mothers’ education showed an important role as well, where families with higher mother literacy had lower infection probabilities. The household UBN measured in our survey also showed an effect increasing infection probabilities at a household level.

**Fig 4:**
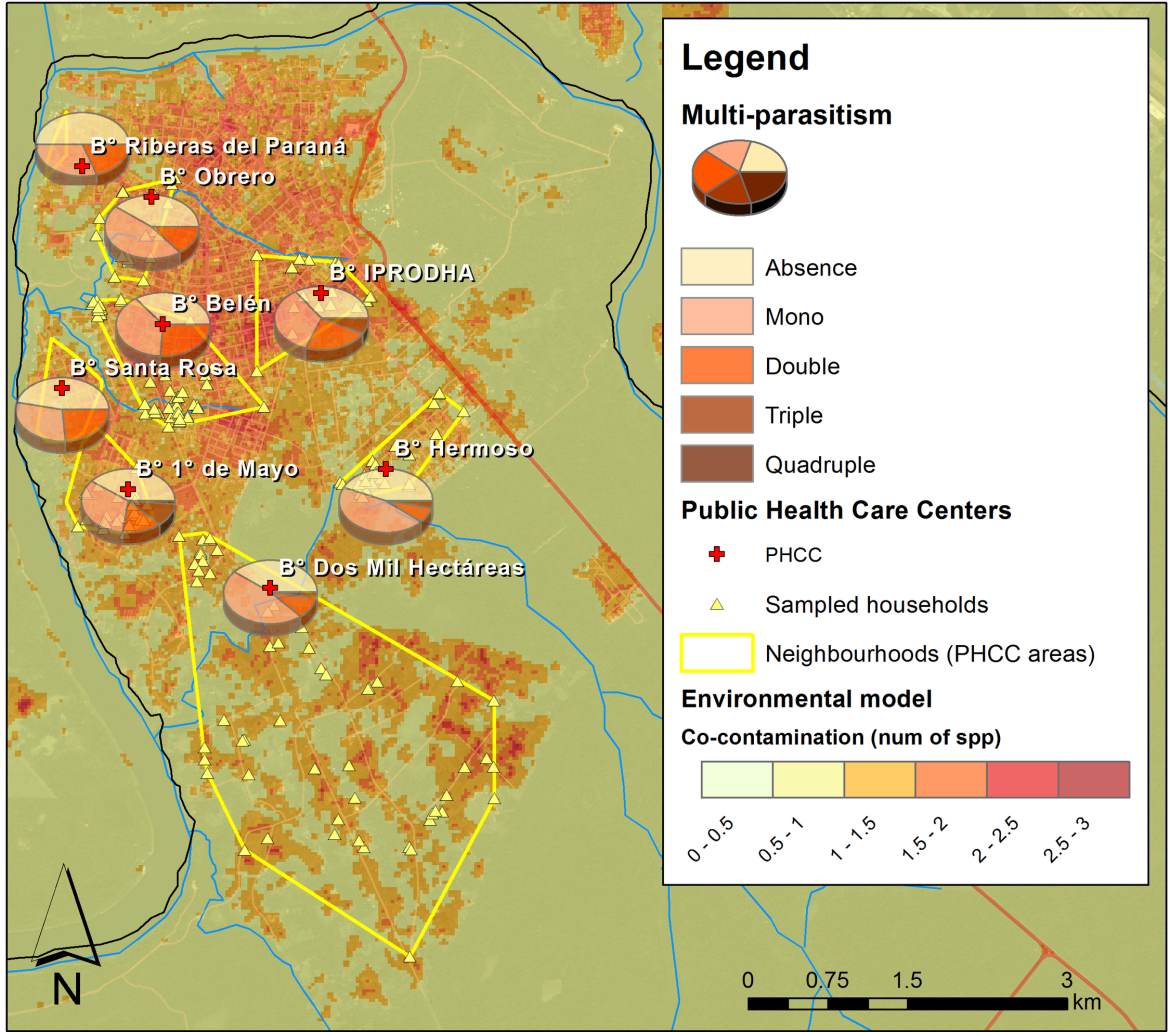
Environmental contamination and children health at a neighborhood scale. The map shows the spatial relationship between the environmental co-contamination predicted at a neighborhood level and the multi-parasitoses found in the children population of each neighborhood. This map was created with ArcGIS 10.4 (www.arcgis.com).

At a regional level (PHCC level), the most important predictor was the co-contamination level in the neighborhood (Fig 3), evidencing higher co-infection of children which live in the more intensively contaminated areas (Fig 4; Table 4). The socioeconomic conditions described by the National Census at a regional level were not good predictors (S9, S10 and S11 Tables).

## Discussion

The present study provides a holistic description of environmental and socio-demographic characteristics associated with the environmental contamination and human infections by multiple enteric parasites. The tri-border area that comprises the Iguazú Municipality shows a high prevalence of parasites both in the environment and in the children population with shared parasite genera among dogs, soil and humans. Importantly, our results provide evidence that parasites pose high impact in terms of environmental contamination and human infection, and that there is a combination of elements acting at different levels which are the responsible for parasite maintenance in the area.

Guiding by WHO recommendation of central actions to perform in areas where information about intestinal parasites infections is absent or is notoriously poor [66], in this work we generated the baseline information about specific and multiple parasites prevalence for the Iguazú tri-border city, including three spheres (environment, animals and humans) as well as the interplay among them. Considering helminths and protozoa, at least sixteen genera were detected in environmental samples while not less than twelve genera we recovered from children feces. We observed shared parasite genera among dogs, soil and humans samples, most of them possessing zoonotic potential.

Parasite prevalence in children was near to sixty per cent, with >30% of multiparasitoses. This evidences a state of situation that requires immediate efforts to improve child health in the area. The pathogen protozoa *G*. *intestinalis* was the most prevalent parasite, followed by *E*. *vermicularis*, *H*. *nana* and hookworms, while *A*. *lumbricoides*, *T*. *trichiura*, and *S*. *stercoralis* were not the predominant enteric parasites in this study. Accordingly, this patter has been reported by several authors in tropical and subtropical areas of eastern South America [35, 67]. *A*. *lumbricoides* presented a more significant role in aboriginal communities of the region [68, 69] suggesting that specific aspects of their socio-environmental condition favor the completion of its life cycle, maintenance and dispersion. Regarding the environmental samples, >70% of the surveyed sites were contaminated. Hookworms were the main pathogens detected at environmental samples followed by *Toxocara* spp, *Trichuris* spp and *Giardia*, the same group of species found in other regions of Misiones province [35, 68]. The high levels of contaminated sites depict an unhealthy frame that contributes to the detriment of communities their environmental, animal and human health.

This study provides a first description of the parasite community occurring in this region of the tri-border among Argentina, Brazil and Paraguay. The high prevalence and diversity of parasites found in both, the environment and in human samples, advocate that species-specific studies should be delineated in this area for a better understanding of the causalities of these parasitoses. For this purpose, it is important to consider for the study design and variables selection the specific life cycles of the described parasites, taking into account the seasonality of some parasite cycles [70] and the potential non-stationary characteristics of some spatial processes [71]. Additionally, it is important to contemplate the multiple sources of uncertainties behind these studies, from the diagnosis to model development, especially when developing spatial predictive models [72]. In spite of these several aspects needed for a better understanding of the parasitoses at the Iguazú region, in this work we provide an important starting point for the comprehending of the numerous factors and levels involved in the persistence of these parasitoses.

Multiple factors can be involved in determining human health. Exploring one factor at a time may limit the holistic understating of the multiple levels and the complexity of the interaction between the diseases and the individuals [8, 9, 29]. This interaction can involve factors related to the individual itself (with its age, nutritional status, etc.), its proximal atmosphere (family composition, household characteristics, etc.), its neighborhood (socio-economic conditions of the area), and the environment potentially acting at different levels (e.g. household or neighborhood). To account for the complexity of factors underlying the parasite transmission, we chose to use a hierarchical approach which allows considering all these levels with the potential multiple factors involved at the same time. The fact that our final models did include multiple factors comprising all these levels, further support the paradigm of an integrative approach for understanding and preventing these diseases.

Considering individual level, age group, playing habits and previous treatment evidenced a significant association with intestinal parasite infection. The age of the host appears as a determinant factor in both, the presence and parasite co-infection. Although scholar-age children were the most parasitized group, children under five years old evidenced high prevalence evidencing the importance of not neglect this group when assessing epidemiology and interventions of children entero-parasitosis [73, 74]. Age-specific playing habits such as playing with soil favor more contact with the routes of transmission of parasite infective stages, and our results showed its importance in maintaining both, presence and co-infection prevalence. Hygiene practices, such as wearing shoes and hand washing, did not show incidence in our models when combined with other factors. Hygiene habits especially related to hand washing are not well ingrained in infant population, being a substantial determinant to parasites acquisition route [75, 76]. However, it has been reported that parents tend to over report hand washing behaviors of their children, which would systematically decrease any apparent benefits and in some cases even shown negative effects [76, 77]. Potential bias associated with socially desirable responses need to be carefully analyzed and the results validated with a methodology that allows direct observation of the issue that investigates.

Another individual factor detected as potential infection determinant is related to children previously treated with antiparasitic drugs. This suggests that the intrinsic or extrinsic conditions that favor these infections remain unsolved. Host traits that may promote higher susceptibility are in close relation with host exposure to risk factors and immune defense [78]. Relative to this, the nutritional status of the children population under study reflected wasting and stunting as the main profiles. In endemic areas, intestinal parasites usually contribute significantly to children stunting impairing growth by malnutrition processes [15, 79]. Although we found a lower prevalence of parasites in overweight and obese children, we did not find an association between parasites presence and co-infection with wasted growth and stunted individuals. These results were also reported by other researchers [17], suggesting that nutritional analysis could require a detailed exploration considering specific parasites effects in order to avoid biases produced when general parasites prevalence are measured. At the same time, synergism with another pathogen of different etiology such as bacteria must also be taken into consideration in this issue [79], making even more complex their interpretation.

The understanding of the parasite prevalence among the children living in Iguazú also meant observing their family and households. Children from families managed by single mothers present higher probabilities of infection, possibly due to the increased family responsibilities for the mother that compromises the educational, health and economic aspects. Mother's literacy is also an important socio-economic factor defining parasite presence and this was described as one of the key factors underpinning their prevalence [12, 18, 23, 80]. Another important finding was that larger families (with more than three children) presented higher co-infection patterns, which is in close relation with overcrowding conditions usually associated with higher prevalence and chronicity of intestinal parasites infections [81, 82].

Considering household deficiencies, water and sanitization are two key components that establish deleterious effects on health outcomes being connected with parasites infections. Therefore, household access to tap water and safe excreta disposal pose central importance for targeted interventions [81, 83, 84]. In Iguazú, the lack of widespread sewerage services in the city depicts in many areas an excreta disposal pattern defined by latrines and septic tank which unfortunately lacks public control and technical advice. Thus, houses without safe excreta disposal presented higher level of co-infection. Certainly, the association of intestinal parasitic to inadequately disposing of excreta is one of the main socio-environmental determinants identified in developing countries [67, 85–88].

Surprisingly, we found that households with tap water had higher probabilities of infected children. We associate this result with the high prevalence of *Giardia intestinalis* we found in the children. The vulnerability of drinking water supply systems to pathogens contamination and the consequent increase of risk of waterborne diseases have been highlighted in several studies [89, 90]. *Giardia* is an anaerobic flagellated protozoa capable of encysting through a complex process of cyst wall formation [91], being this infective form resistant to common disinfection controls such as chlorine and chloramines [92]. In order to fill data gaps and provide technical assistance in this region, a comprehensive monitoring strategy that incorporates and links water quality data and human disease data in a spatial database are needed.

At the household level, our UBN categorization was a good predictor of parasite infection in the children of these houses. UBS local assessment possesses a high programmatic value to guide public support policies improving housing infrastructure to address health issues. We observed that socio-demographic data obtained from census blocks or districts (i.e. our UBN estimated at a neighborhood scale from National Census data) was not able to describe the most proximal conditions associated with parasite infections, suggesting that socio-demographic data need to be conceived at household level especially in areas lacking uniform territorial organization. Accordingly, Karagiannis-Voules *et al*. [93] found that combining data from different scales did not evidence the heterogeneity that may exist in a community when they used socioeconomic proxies at large scales trying to explain soil-transmitted helminth infections at household level in Cambodia.

Remarkably, individual and household characteristics were not enough to explain completely the patterns of parasite infection we found in the children of Iguazú, and the inclusion of environmental factors at a larger scale was necessary. Neighborhoods with higher co-contamination levels in the environment presented higher co-infection in their children population (Fig 3) and this was important to understand and predict the multi-parasitism we found in each child. Environmental fecal contamination (from human and animal origin) has been studying from different perspectives considering aspects linked with agricultural practices, anthropogenic activities, urbanization, land use patterns and economics concern. In all of these issues, exposure to pathogens and its implication in public health have been highly emphasized [81, 94, 95]. These parasites raise the soil throughout infected excretes, therefore, the incidence and prevalence of intestinal parasites in the environment are taken as indicators of fecal contamination to which animals and humans are exposed [88].

On the one hand, the clear spatial relationship we found between the environmental contamination and the co-infection of children at a neighborhood level suggests that this approach of spatial explicit environmental assessment could provide an important shortcut in the field work to then address more effective public health interventions. Furthermore, this emphasizes that the solutions to this type of diseases require high community commitment as well as political management which mediate structural and sanitary solutions in the whole neighborhoods composing the city. On the other hand, our multi-level models combined with household level socio-economical information can be a very powerful tool for predicting potential parasite infection and co-infection to localize areas for more specific interventions.

### Conclusions

Our work represents the major survey of intestinal parasites in human and environmental samples developed in the region, providing useful benchmark information for prioritizing and enlightening targeting of interventions. One important finding of our work is the significance of considering multi-level determinants for understanding the maintenance and propagation of intestinal parasites in a sensitive population of Argentina. Our results show that environmental surveys could guide human surveys and interventions on a neighborhood level, but simultaneously, the attention of socio-economic conditions at the household level, and the individual child care are of great relevance. The capacity of combining environmental and human field surveys to identify key component acting in different levels enhances the potential of using the new understanding and tools to struggle these neglected tropical diseases.

## Supporting information

S1 Fig Confidence intervals for co-contamination risk maps. Maps representing the co-contamination level predicted by the final model for the environmental contamination by parasites showing a) the lower 95% confidence interval; b) the final model; c) the upper 95% confidence interval.
(TIF)

S1 Table Landscape scale variables. List of variables utilized for describing the environmental conditions at a landscape scale.
(DOCX)

S2 Table Local scale variables. List of variables utilized for describing the environmental conditions at a local scale.
(DOCX)

S3 Table Individual level variables. List of variables utilized for describing the children conditions at the individual level.
(DOCX)

S4 Table Household level variables. List of variables utilized for describing the household conditions.
(DOCX)

S5 Table PHCC level variables. List of variables utilized for describing the conditions of the PHCC area.
(DOCX)

S6 Table Environment univariate analysis. Univariate GLM developed for predicting parasite contamination (left) and co-contamination (right) in the environment at the Iguazú area. Selected variables for representing each group in further model combinations and model selection are in bold.
(DOCX)

S7 Table Model selection for parasite presence in the environment. Summary of the model selection procedure for the environmental risk assessment for predicting the presence of parasites at the Iguazú Municipality.
(DOCX)

S8 Table Model selection for co-contamination in the environment. Summary of the model selection procedure for the environmental risk assessment for predicting the parasite co-contamination (number of species of parasites) at the Iguazú Municipality.
(DOCX)

S9 Table Children univariate analysis. Univariate GLM developed for predicting parasite presence (left) and co-infection (right) in the child population of the Iguazú area. Selected variables for representing each group in further model combinations and model selection are in bold.
(DOCX)

S10 Table Model selection for children infection with parasites. Summary of the mixed effects model selection procedure for selecting the best parsimonious model for predicting parasite infection in the children population of Iguazú area. The Δ column depicts the difference between a model’s Akaike’s Information Criterion (AIC) and that of the best-fitting model.
(DOCX)

S11 Table Model selection for predicting children multi-parasitoses. Summary of the mixed effects model selection procedure for selecting the best parsimonious model for predicting co-infection level in the children population of Iguazú area. The Δ column depicts the difference between a model’s Akaike’s Information Criterion (AIC) and that of the best-fitting model.
(DOCX)
