# Supplementary figures and images for: Environmental and socio-demographic individual, family and neighborhood factors associated with children intestinal parasitoses at Iguazú, in the subtropical northern border of Argentina
Source: PLoS Negl Trop Dis. 2017 Nov 20;11(11):e0006098. doi: 10.1371/journal.pntd.0006098 (PMC5714390; doi:10.1371/journal.pntd.0006098)

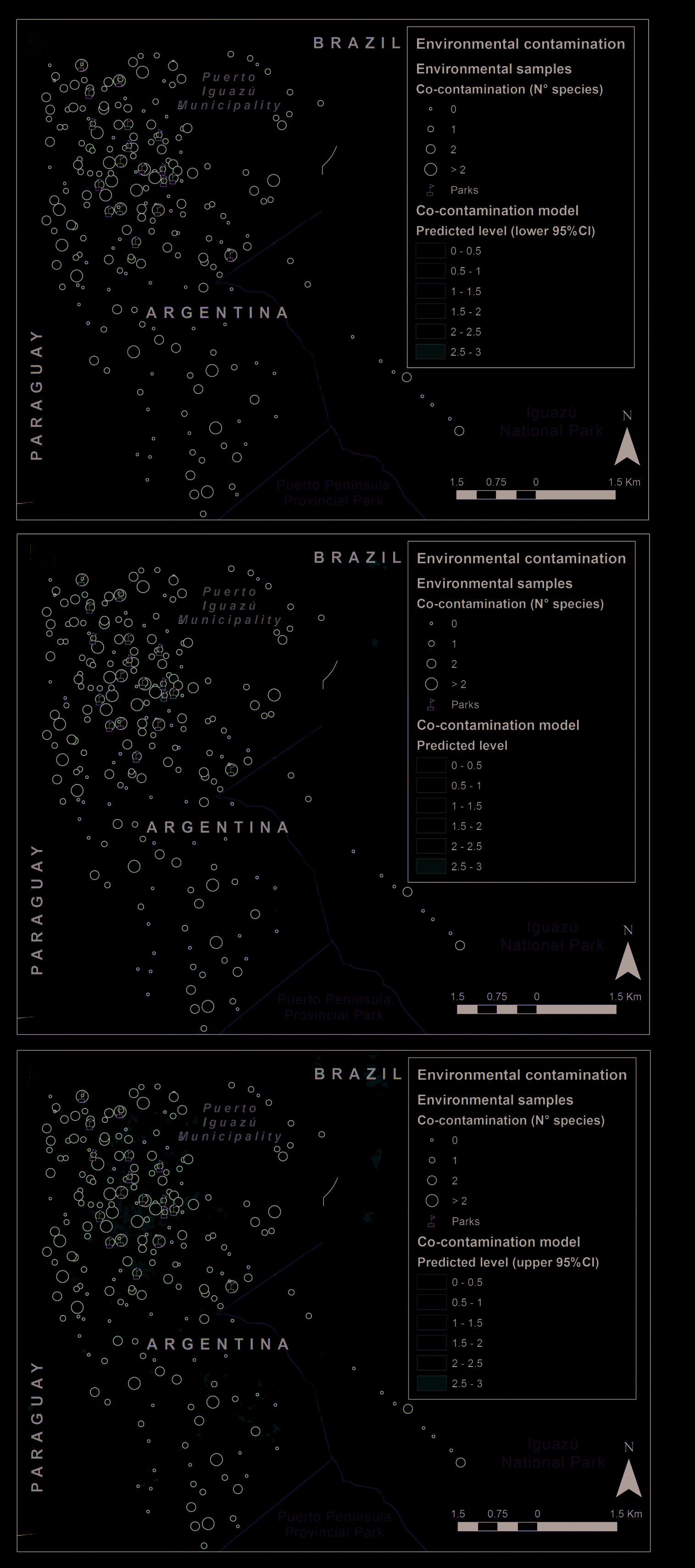

Supplement: S1 Fig — Maps representing the co-contamination level predicted by the final model for the environmental contamination by parasites showing a) the lower 95% confidence interval; b) the final model; c) the upper 95% confidence interval. (TIF) [file pntd.0006098.s001.tif]
