# Supplementary material for: Environmental and socio-demographic individual, family and neighborhood factors associated with children intestinal parasitoses at Iguazú, in the subtropical northern border of Argentina
Source: PLoS Negl Trop Dis. 2017 Nov 20;11(11):e0006098. doi: 10.1371/journal.pntd.0006098 (PMC5714390; doi:10.1371/journal.pntd.0006098)
Supplement: S1 Table — List of variables utilized for describing the environmental conditions at a landscape scale. (DOCX) [file pntd.0006098.s002.docx]

**S1 Table. Landscape scale variables.** List of variables utilized for describing the environmental conditions at a landscape scale.

| **Group of variables** | **Name** | **Type** | **Description** | **Source** |
| --- | --- | --- | --- | --- |
| **Topography** | Distance to rivers | Continuous | Distance to the closest river or stream (m) | Developed from local GIS database |
|  | Elevation | Continuous | Elevation (m osl) | Calculated from ASTER DEM: <https://asterweb.jpl.nasa.gov/gdem.asp> |
|  | Slope | Continuous | Slope of the terrain (%) |  |
|  | Orientation | Categorical | Aspect of the terrain (orientation: N, E, S, W) |  |
| **Social and economic conditions** | Street density | Continuous | Streets density measured as the number of 1-m cells crossed by streets in a circle of 100-m radius. | Open street maps ([www.openstreetmap.org](http://www.openstreetmap.org)) – Date of access: November 22^th^, 2014. |
|  | Population density | Continuous | Population density (inhabitants/km^2^). | Measured for census districts by the National Census of [INDEC [1](#_ENREF_1)]. |
|  | Inadequate services | Continuous | Percentage of houses with inadequate public services connections. |  |
|  | Overcrowding | Continuous | Percentage of houses with overcrowding conditions. |  |
|  | UBN | Continuous | Percentage of houses with unsatisfied basic needs. |  |
| **Land cover** | Trees | Continuous | Percentage of tree cover in a circle of 30-m radius. | Google Earth image classification (date of source image November 3th, 2014 - <https://www.google.com/earth>). Classified using a supervised Maximum Likelihood classification with ArcGIS 10.4. |
|  | Grass | Continuous | Percentage of grasslands in a circle of 30-m radius. |  |
|  | Bare soil | Continuous | Percentage of bare soil in a circle of 30-m radius. |  |
|  | Construction | Continuous | Percentage of constructed area in a circle of 30-m radius. |  |
|  | Surface temperature | Continuous | Surface temperature (estimated in a 60-m radius circle) estimated from Landsat 8 satellite images and land cover. Higher temperatures are associated with more urbanized areas [[2](#_ENREF_2)] | Landsat 8 ETM (date: August 8^th^, 2014) / Google Earth classification / calculation description at: <http://landsat.usgs.gov/Landsat8_Using_Product.php> |

# References mentioned in the supplementary materials

1. Censo Nacional de Población y Vivienda 2010 [database on the Internet]. Instituto Nacional de Estadística y Censo de Argentina. 2010 [cited 15 Jun 2014]. Available from: [www.indec.mecon.gov.ar](http://www.indec.mecon.gov.ar).

2. Carlson TN, Traci Arthur S. The impact of land use — land cover changes due to urbanization on surface microclimate and hydrology: a satellite perspective. Global and Planetary Change. 2000;25(1–2):49-65.
