# Supplementary material for: Environmental and socio-demographic individual, family and neighborhood factors associated with children intestinal parasitoses at Iguazú, in the subtropical northern border of Argentina
Source: PLoS Negl Trop Dis. 2017 Nov 20;11(11):e0006098. doi: 10.1371/journal.pntd.0006098 (PMC5714390; doi:10.1371/journal.pntd.0006098)
Supplement: S2 Table — List of variables utilized for describing the environmental conditions at a local scale. (DOCX) [file pntd.0006098.s003.docx]

**S2 Table.** **Local scale variables.** List of variables utilized for describing the environmental conditions at a local scale.

| **Group of variables** | **Name** | **Type** | **Description** | **Source** |
| --- | --- | --- | --- | --- |
| **Local scale** | Presence of dog | Binary | The presence of dogs in the area. | Information collected in a visual survey made on the site and at the moment of the environmental sample collection. |
|  | Presence of farm animals | Binary | The presence of farm animals in the area. |  |
|  | Trash | Binary | The presence of trash in the area. |  |
|  | Substrate | Categorical | Street/area main substrate (dirt road, paved, asphalt). |  |
|  | Latrine | Binary | The presence of latrine in the houses of the area. |  |
