# Supplementary material for: Environmental and socio-demographic individual, family and neighborhood factors associated with children intestinal parasitoses at Iguazú, in the subtropical northern border of Argentina
Source: PLoS Negl Trop Dis. 2017 Nov 20;11(11):e0006098. doi: 10.1371/journal.pntd.0006098 (PMC5714390; doi:10.1371/journal.pntd.0006098)
Supplement: S3 Table — List of variables utilized for describing the children conditions at the individual level. (DOCX) [file pntd.0006098.s004.docx]

**S3 Table.** **Individual level variables.** List of variables utilized for describing the children conditions at the individual level.

| **Group of variables** | **Name** | **Type** | **Description** | **Source** |
| --- | --- | --- | --- | --- |
| **Individual** | Sex | Binary | Male or female | Pre-tested and structured questionnaire (see Methods) |
|  | Age group | Ordinal | 1: under 5 years old; 2: between 5 to 9 years old; 3: over 9 years old |  |
| **Nutritional conditions** | Wasted | Binary | Weight for length/height Z-score (WHZ) < -2 SD | Data calculated from the children measurements collected by the local authorities at each PHCC. Calculations were done using the sex-specific WHO Child Growth Standards through WHO Anthro and WHO Anthro Plus software. |
|  | Stunted | Binary | Length/height for age Z-score (HAZ) < -2 SD |  |
|  | Underweight | Binary | Weight for age Z-score (WAZ) < -2 SD |  |
|  | Obese or overweight | Binary | Body mass for age Z-score (BMIZ) > 1 SD |  |
| **Exposure** | Previous deworming treatment | Binary | Children who received previous antiparasitic treatment | Pre-tested and structured questionnaire (see Methods) |
| **Habits** | Hand washing | Binary | Child with hand washing habit | Pre-tested and structured questionnaire (see Methods) |
|  | Using shoes | Binary | Child with the habit of wearing shoes |  |
|  | Playing with soil | Binary | Child with the habit of playing with soil |  |
