# Supplementary material for: Environmental and socio-demographic individual, family and neighborhood factors associated with children intestinal parasitoses at Iguazú, in the subtropical northern border of Argentina
Source: PLoS Negl Trop Dis. 2017 Nov 20;11(11):e0006098. doi: 10.1371/journal.pntd.0006098 (PMC5714390; doi:10.1371/journal.pntd.0006098)
Supplement: S4 Table — List of variables utilized for describing the household conditions. (DOCX) [file pntd.0006098.s005.docx]

**S4 Table.** **Household level variables.** List of variables utilized for describing the household conditions.

| **Group of variables** | **Name** | **Type** | **Description** | **Source** |
| --- | --- | --- | --- | --- |
| **House** | Economic status | Ordinal | The number of main components of the dwelling (ceiling, floor or walls) made of cement (e.g. level 3 = all made of cement). | Pre-tested and structured questionnaire (see Methods) |
|  | Unsatisfied Basic Needs (UBN) | Binary | The presence of UBN according to the criteria of the National Institute of Statistics and Censuses surveyed at the household level. |  |
| **Yard** | Peridomiciliary hygiene | Binary | Presence of trash around households | Pre-tested and structured questionnaire (see Methods) |
|  | Farm animals | Binary | Presence of farm animals in the yard |  |
| **Food** | Origin of vegetables | Categorical | Main origin of the vegetables consumed by the family: homegrown vegetables, neighborhood's grocery store, supermarket, regional fair, or no vegetable consumption | Pre-tested and structured questionnaire (see Methods) |
| **WASH** | Tap water | Binary | Household with access to tap water | Pre-tested and structured questionnaire (see Methods) |
|  | Safe excreta disposal | Binary | The house possesses a toilet and septic tank. |  |
|  | Safe waste disposal | Binary | Municipal waste collection |  |
| **Family** | Children per family | Continuous | Number of children per family | Pre-tested and structured questionnaire (see Methods) |
|  | Large family | Binary | Families with more than 3 children |  |
|  | Young mother | Binary | Mother under 30 years old |  |
|  | Single mother | Binary | Single mother as head of household |  |
|  | Mother literacy | Binary | Mother with primary school complete |  |
|  | Working mother | Binary | Mother working outside the home |  |
|  | Overcrowding | Binary | More than three children per room in the house |  |
| **Pests** | Rodents | Binary | Presence of rodents in the house | Pre-tested and structured questionnaire |
| **Environmental risk** (household area) | Co-contamination | Continuous | The co-contamination level predicted for the household area by the environmental models | Models developed in this work (see Methods and Results). |
