# Supplementary material for: Environmental and socio-demographic individual, family and neighborhood factors associated with children intestinal parasitoses at Iguazú, in the subtropical northern border of Argentina
Source: PLoS Negl Trop Dis. 2017 Nov 20;11(11):e0006098. doi: 10.1371/journal.pntd.0006098 (PMC5714390; doi:10.1371/journal.pntd.0006098)
Supplement: S5 Table — List of variables utilized for describing the conditions of the PHCC area. (DOCX) [file pntd.0006098.s006.docx]

**S5 Table.** **PHCC level variables** List of variables utilized for describing the conditions of the PHCC area.

| **Group of variables** | **Name** | **Type** | **Description** | **Source** |
| --- | --- | --- | --- | --- |
| **Socio-economic** | Water supply | Continuous | Mean percentage of houses with water supply in the neighborhood | Measured for census districts by the National Census of [INDEC [1](#_ENREF_1)]. |
|  | Water service | Continuous | Mean percentage of houses with public water service in the neighborhood |  |
|  | Population density | Continuous | Mean population density (ind/km^2^) in the neighborhood |  |
|  | Unsatisfied basic needs | Continuous | Mean percentage of houses with unsatisfied basic needs in the neighborhood |  |
| **Environmental risk** | Co-contamination | Continuous | Mean co-contamination level predicted at PHCC level by the environmental models. | Models developed in this work. |

# References

1. Censo Nacional de Población y Vivienda 2010 [database on the Internet]. Instituto Nacional de Estadística y Censo de Argentina. 2010 [cited 15 Jun 2014]. Available from: [www.indec.mecon.gov.ar](http://www.indec.mecon.gov.ar).
