# Supplementary material for: Environmental and socio-demographic individual, family and neighborhood factors associated with children intestinal parasitoses at Iguazú, in the subtropical northern border of Argentina
Source: PLoS Negl Trop Dis. 2017 Nov 20;11(11):e0006098. doi: 10.1371/journal.pntd.0006098 (PMC5714390; doi:10.1371/journal.pntd.0006098)
Supplement: S6 Table — Univariate GLM developed for predicting parasite contamination (left) and co-contamination (right) in the environment at the Iguazú area. Selected variables for representing each group in further model combinations and model selection are in bold. (DOCX) [file pntd.0006098.s007.docx]

**S6 Table. Environment univariate analysis.** Univariate GLM developed for predicting parasite contamination (left) and co-contamination (right) in the environment at the Iguazú area. Selected variables for representing each group in further model combinations and model selection are in bold.

| **Group of variables** | **Variable** | **Presence of contamination** | | | | **Co-contamination** | | | |
| --- | --- | --- | --- | --- | --- | --- | --- | --- | --- |
|  |  | **Estimate** | **Std. Error** | **z value** | **p** | **Estimate** | **Std. Error** | **z value** | **p** |
| **Landscape scale** |  |  |  |  |  |  |  |  |  |
| Topography | **Elevation** | **-0.015** | **0.008** | **-2.048** | **0.041** | **-0.006** | **0.003** | **-1.869** | **0.062** |
|  | Distance to rivers | -0.001 | 0.000 | -1.237 | 0.216 | 0.000 | 0.000 | -1.106 | 0.269 |
|  | Slope | 0.103 | 0.089 | 1.156 | 0.248 | 0.012 | 0.029 | 0.406 | 0.685 |
|  | Orientation (East + intercept) |  |  |  |  |  |  |  |  |
|  | (North) | 0.254 | 0.569 | 0.447 | 0.655 | 0.259 | 0.223 | 1.158 | 0.247 |
|  | (South) | -0.105 | 0.593 | -0.176 | 0.860 | 0.040 | 0.250 | 0.162 | 0.872 |
|  | (West) | -0.174 | 0.503 | -0.347 | 0.729 | 0.148 | 0.210 | 0.704 | 0.481 |
| Social and economic conditions | **Street density** | **0.019** | **0.006** | **3.224** | **0.001** | **0.008** | **0.002** | **3.623** | **<0.001** |
|  | Population density | 0.000 | 0.000 | 2.238 | 0.025 | 0.000 | 0.000 | 2.909 | 0.004 |
|  | Inadequate services | -1.944 | 0.680 | -2.861 | 0.004 | -0.613 | 0.208 | -2.951 | 0.003 |
|  | Overcrowding | -1.803 | 0.976 | -1.847 | 0.065 | -1.339 | 0.451 | -2.970 | 0.003 |
|  | UBN | -1.491 | 0.849 | -1.757 | 0.079 | -1.148 | 0.359 | -3.197 | 0.001 |
| Land cover | Trees | -0.020 | 0.008 | -2.561 | 0.010 | -0.013 | 0.004 | -3.368 | 0.001 |
|  | Grass | 0.010 | 0.019 | 0.555 | 0.579 | -0.004 | 0.007 | -0.517 | 0.605 |
|  | Bare soil | 0.019 | 0.008 | 2.258 | 0.024 | 0.011 | 0.003 | 3.546 | <0.001 |
|  | Construction | 0.026 | 0.015 | 1.735 | 0.083 | 0.010 | 0.005 | 1.932 | 0.053 |
|  | **Surface temperature** | **0.259** | **0.084** | **3.085** | **0.002** | **0.144** | **0.035** | **4.177** | **<0.001** |

**S6 Table. Environment univariate analysis.** (Cont.)

| **Group of variables** | **Variable** | **Presence of contamination** | | | | **Co-contamination** | | | |
| --- | --- | --- | --- | --- | --- | --- | --- | --- | --- |
|  |  | **Estimate** | **Std. Error** | **z value** | **p** | **Estimate** | **Std. Error** | **z value** | **p** |
| **Local scale** |  |  |  |  |  |  |  |  |  |
|  | Presence of dogs | 0.856 | 0.523 | 1.638 | 0.101 | 0.425 | 0.278 | 1.527 | 0.127 |
|  | Presence of farm animals | -0.576 | 0.367 | -1.571 | 0.116 | -0.163 | 0.148 | -1.105 | 0.269 |
|  | **Trash** | **0.904** | **0.371** | **2.436** | **0.015** | **0.356** | **0.170** | **2.089** | **0.037** |
|  | Substrate (dirt + intercept) |  |  |  |  |  |  |  |  |
|  | (paved) | 0.511 | 0.690 | 0.740 | 0.459 | 0.108 | 0.267 | 0.405 | 0.686 |
|  | (asphalt) | 0.657 | 0.472 | 1.392 | 0.164 | 0.325 | 0.167 | 1.949 | 0.051 |
|  | Latrine | -0.764 | 0.362 | -2.110 | 0.035 | -0.287 | 0.151 | -1.907 | 0.057 |
|  | Houses characteristics (bricks + int.) |  |  |  |  |  |  |  |  |
|  | (wood) | -0.489 | 0.442 | -1.107 | 0.268 | -0.148 | 0.184 | -0.806 | 0.420 |
|  | (cement) | 0.033 | 0.515 | 0.064 | 0.949 | 0.178 | 0.193 | 0.924 | 0.356 |
