# Supplementary material for: Environmental and socio-demographic individual, family and neighborhood factors associated with children intestinal parasitoses at Iguazú, in the subtropical northern border of Argentina
Source: PLoS Negl Trop Dis. 2017 Nov 20;11(11):e0006098. doi: 10.1371/journal.pntd.0006098 (PMC5714390; doi:10.1371/journal.pntd.0006098)
Supplement: S7 Table — Summary of the model selection procedure for the environmental risk assessment for predicting the presence of parasites at the Iguazú Municipality. (DOCX) [file pntd.0006098.s008.docx]

**S7 Table.** **Model selection for parasite presence in the environment.** Summary of the model selection procedure for the environmental risk assessment for predicting the presence of parasites at the Iguazú Municipality.

| **Model** | **Variable groups** | **Fixed variables** | **logLink** | **AICc** | **Delta AICc** |
| --- | --- | --- | --- | --- | --- |
| ME11 | Social and economic conditions + Local conditions | Streets density + Trash | -86.614 | 179.4 | 0 |
| ME13 | Land cover + Local conditions | Surface temperature + Trash | -86.707 | 179.6 | 0.18 |
| ME15 | Social and economic conditions + Land cover + Local conditions | Streets density + Surface temperature + Trash | -85.699 | 179.7 | 0.28 |
| ME14 | Topography + Land cover + Local conditions | Elevation + Surface temperature + Trash | -86.350 | 181.0 | 1.58 |
| ME12 | Topography + Social and economic conditions + Local conditions | Elevation + Streets density + Trash | -86.598 | 181.5 | 2.08 |
| ME16 | Topography + Social and economic conditions + Land cover + Local conditions | Elevation + Streets density + Surface temperature + Trash | -85.649 | 181.7 | 2.32 |
| ME09 | Local conditions | Trash | -89.380 | 182.8 | 3.45 |
| ME10 | Topography + Local conditions | Elevation + Trash | -88.755 | 183.7 | 4.28 |
| ME03 | Social and economic conditions | Streets density | -89.815 | 183.7 | 4.32 |
| ME05 | Land cover | Surface temperature | -90.060 | 184.2 | 4.81 |
| ME07 | Social and economic conditions + Land cover | Streets density + Surface temperature | -89.133 | 184.4 | 5.04 |
| ME06 | Topography + Land cover | Elevation + Surface temperature | -89.755 | 185.7 | 6.28 |
| ME04 | Topography + Social and economic conditions | Elevation + Streets density | -89.797 | 185.8 | 6.37 |
| ME08 | Topography + Social and economic conditions + Land cover | Elevation + Streets density + Surface temperature | -89.095 | 186.5 | 7.07 |
| ME01 | Null model | Null model | -92.337 | 186.7 | 7.31 |
| ME02 | Topography | Elevation | -91.739 | 187.6 | 8.17 |
