# Supplementary material for: Environmental and socio-demographic individual, family and neighborhood factors associated with children intestinal parasitoses at Iguazú, in the subtropical northern border of Argentina
Source: PLoS Negl Trop Dis. 2017 Nov 20;11(11):e0006098. doi: 10.1371/journal.pntd.0006098 (PMC5714390; doi:10.1371/journal.pntd.0006098)
Supplement: S8 Table — Summary of the model selection procedure for the environmental risk assessment for predicting the parasite co-contamination (number of species of parasites) at the Iguazú Municipality. (DOCX) [file pntd.0006098.s009.docx]

**S8 Table.** **Model selection for co-contamination with parasites in the environment.** Summary of the model selection procedure for the environmental risk assessment for predicting the co-contamination (number of species of parasites) at the Iguazú Municipality.

| **Model** | **Variable groups** | **Fixed variables** | **logLink** | **AICc** | **Delta AICc** |
| --- | --- | --- | --- | --- | --- |
| ME13 | Land cover + Local conditions | Surface temperature + Trash | -203.211 | 412.6 | 0 |
| ME15 | Social and economic conditions + Local conditions | Streets density + Trash | -203.012 | 414.3 | 1.7 |
| ME14 | Topography + Land cover + Local conditions | Elevation + Surface temperature + Trash | -203.091 | 414.5 | 1.9 |
| ME05 | Land cover | Surface temperature | -205.754 | 415.6 | 3.0 |
| ME16 | Topography + Social and economic conditions + Land cover + Local conditions | Elevation + Streets density + Surface temperature + Trash | -202.97 | 416.4 | 3.8 |
| ME07 | Social and economic conditions + Land cover | Streets density + Surface temperature | -205.498 | 417.2 | 4.6 |
| ME06 | Topography + Land cover | Elevation + Surface temperature | -205.585 | 417.3 | 4.8 |
| ME11 | Social and economic conditions + Local conditions | Streets density + Trash | -205.866 | 417.9 | 5.3 |
| ME08 | Topography + Social and economic conditions + Land cover | Elevation + Streets density + Surface temperature | -205.438 | 419.1 | 6. 6 |
| ME12 | Topography + Social and economic conditions + Local conditions | Elevation + Streets density + Trash | -205.832 | 419.9 | 7.4 |
| ME03 | Social and economic conditions | Streets density | -208.22 | 420.5 | 7.9 |
| ME09 | Local conditions | Trash | -208.477 | 421.0 | 8.5 |
| ME10 | Topography + Local conditions | Elevation + Trash | -207.835 | 421.8 | 9.3 |
| ME04 | Topography + Social and economic conditions | Elevation + Streets density | -208.188 | 422.5 | 9.9 |
| ME01 | Null model | Null model | -210.785 | 423.6 | 11.0 |
| ME02 | Topography | Elevation | -210.169 | 424.4 | 11.8 |
