# Supplementary material for: Environmental and socio-demographic individual, family and neighborhood factors associated with children intestinal parasitoses at Iguazú, in the subtropical northern border of Argentina
Source: PLoS Negl Trop Dis. 2017 Nov 20;11(11):e0006098. doi: 10.1371/journal.pntd.0006098 (PMC5714390; doi:10.1371/journal.pntd.0006098)
Supplement: S9 Table — Univariate GLM developed for predicting parasite presence (left) and co-infection (right) in the child population of the Iguazú area. Selected variables for representing each group in further model combinations and model selection are in bold. (DOCX) [file pntd.0006098.s010.docx]

**S9 Table.** **Children univariate analysis.** Univariate GLM developed for predicting parasite presence (left) and co-infection (right) in the child population of the Iguazú area. Selected variables for representing each group in further model combinations and model selection are in bold.

| **Group of variables** | **Variables** | **Prevalence** | | | | | **Co-infection** | | | |
| --- | --- | --- | --- | --- | --- | --- | --- | --- | --- | --- |
|  |  | **Estimate** | **Std. Error** | **z value** | **p** | **Estimate** | | **Std. Error** | **z value** | **p** |
| **Individual level** |  |  |  |  |  |  | |  |  |  |
| Child | Sex | -0.171 | 0.200 | -0.855 | 0.392 | -0.022 | | 0.107 | -0.204 | 0.838 |
|  | Age group (gr 1 + intercept) |  |  |  |  |  | |  |  |  |
|  | (group 2) | **0.970** | **0.237** | **4096.000** | **<0.001** | **0.497** | | **0.118** | **4223** | **<0.001** |
|  | (group 3) | -0.088 | 0.256 | -0.344 | 0.731 | 0.174 | | 0.146 | 1191 | 0.233 |
| Nutritional conditions | Wasted | 0.261 | 0.552 | 0.474 | 0.635 | 0.003 | | 0.252 | 0.012 | 0.990 |
|  | Stunted | -0.097 | 0.353 | -0.274 | 0.784 | -0.179 | | 0.177 | -1012 | 0.311 |
|  | Underweight | -0.313 | 0.289 | -1085 | 0.278 | -0.048 | | 0.162 | -0.298 | 0.766 |
|  | Obese or overweight | **-13243** | **0.318** | **-4164** | **<0.001** | **-0.294** | | **0.150** | **-1957** | **0.050** |
| Exposure | Previous deworming treatment | 0.645 | 0.293 | 2205 | 0.028 | 0.051 | | 0.115 | 0.442 | 0.658 |
| Habits | Hand washing | -0.057 | 0.374 | -0.152 | 0.879 | 0.144 | | 0.115 | 1249 | 0.212 |
|  | Wearing shoes | **17858** | **0.413** | **4323** | **<0.001** | 0.051 | | 0.113 | 0.454 | 0.650 |
|  | Playing with soil | 0.562 | 0.301 | 1870 | 0.062 | 0.202 | | 0.117 | 1720 | 0.085 |
| **Household level** |  |  |  |  |  |  | |  |  |  |
| House | Econ. status (level 0 + int.) |  |  |  |  |  | |  |  |  |
|  | (level 1) | 0.512 | 0.511 | 1002 | 0.316 | 0.029 | | 0.147 | 0.195 | 0.846 |
|  | (level 2) | -0.183 | 0.607 | -0.302 | 0.763 | -0.152 | | 0.183 | -0.828 | 0.408 |
|  | (level 3) | -0.195 | 13051 | -0.149 | 0.881 | 0.000 | | 0.385 | -0.001 | 0.999 |
|  | UBN | **18654** | **0.642** | **2905** | **0.004** | **0.519** | | **0.217** | **2397** | **0.017** |

**S9 Table.** **Children univariate analysis.** Continuation.

| **Group of variables** | **Variables** | **Prevalence** | | | | **Co-infection** | | | |
| --- | --- | --- | --- | --- | --- | --- | --- | --- | --- |
|  |  | **Estimate** | **Std. Error** | **z value** | **p** | **Estimate** | **Std. Error** | **z value** | **p** |
| **Household level** |  |  |  |  |  |  |  |  |  |
| Yard | Peridomiciliary hygiene | 0.059 | 0.381 | 0.154 | 0.878 | -0.069 | 0.120 | -0.573 | 0.567 |
|  | Farm animals | 0.362 | 0.454 | 0.798 | 0.425 | 0.032 | 0.135 | 0.239 | 0.811 |
| Diet | Origin of veg. (hmegr. + int0) |  |  |  |  |  |  |  |  |
|  | (grocery store) | -10039 | 0.681 | -1475 | 0.140 | -0.173 | 0.178 | -0.976 | 0.329 |
|  | (supermarket) | **-16550** | **0.747** | **-2216** | **0.027** | **-0.479** | **0.208** | **-2304** | **0.021** |
|  | (regional fair) | -0.943 | 10575 | -0.892 | 0.372 | -0.201 | 0.296 | -0.680 | 0.497 |
|  | (no vegetables) | -23428 | 12785 | -1832 | 0.067 | -0.448 | 0.408 | -1098 | 0.272 |
| WASH | Tap water | 0.181 | 0.395 | 0.457 | 0.647 | 0.066 | 0.116 | 0.572 | 0.568 |
|  | Safe excreta disposal | -0.697 | 0.393 | -1771 | 0.077 | **-0.337** | **0.110** | **-3055** | **0.002** |
|  | Safe waste disposal | -0.695 | 0.392 | -1774 | 0.076 | -0.134 | 0.113 | -1185 | 0.236 |
| Family | Children per family | **0.302** | **0.148** | **2047** | **0.041** | **0.122** | **0.002** | **49.140** | **<0.001** |
|  | Large family | 0.759 | 0.432 | 1755 | 0.079 | **0.413** | **0.116** | **3563** | **<0.001** |
|  | Young mother | 0.302 | 0.391 | 0.773 | 0.440 | 0.050 | 0.114 | 0.438 | 0.661 |
|  | Single mother | 0.847 | 0.514 | 1650 | 0.099 | 0.127 | 0.141 | 0.897 | 0.370 |
|  | Mother literacy | **-0.847** | **0.406** | **-2.086** | **0.037** | -0.208 | 0.114 | -1.827 | 0.068 |
|  | Working mother | 0.019 | 0.527 | 0.036 | 0.971 | -0.105 | 0.161 | -0.653 | 0.514 |
|  | Overcrowding | **0.360** | **0.142** | **2528** | **0.012** | **0.091** | **0.036** | **2541** | **0.011** |
| Pests | Rodents | -0.328 | 0.393 | -0.835 | 0.404 | -0.079 | 0.115 | -0.684 | 0.494 |
| Environmental risk (household) | Co-contamination | 0.476 | 0.716 | 0.670 | 0.503 | 0.194 | 0.212 | 0.912 | 0.362 |
| **PHCC level** |  |  |  |  |  |  |  |  |  |
| Socio-economic  (census data) | Water supply | 0.752 | 1.297 | 0.580 | 0.562 | 0.262 | 0.387 | 0.679 | 0.497 |
|  | Water service | 0.411 | 0.621 | 0.661 | 0.508 | 0.143 | 0.189 | 0.760 | 0.447 |
|  | Population density | 0.0001 | 0.00005 | 1.059 | 0.290 | 0.0003 | 0.00003 | 1.181 | 0.237 |
|  | Unsatisfied basic needs | -1.062 | 1.883 | -0.564 | 0.573 | -0.332 | 0.547 | -0.607 | 0.544 |
| Environmental risk (PHCC) | Parasite contamination | 2.266 | 2.240 | 1.012 | 0.312 | 0.976 | 0.662 | 1475 | 0.140 |
