# Supplementary material for: Environmental and socio-demographic individual, family and neighborhood factors associated with children intestinal parasitoses at Iguazú, in the subtropical northern border of Argentina
Source: PLoS Negl Trop Dis. 2017 Nov 20;11(11):e0006098. doi: 10.1371/journal.pntd.0006098 (PMC5714390; doi:10.1371/journal.pntd.0006098)
Supplement: S10 Table — Summary of the mixed effects model selection procedure for selecting the best parsimonious model for predicting parasite infection in the children population of Iguazú area. The Δ column depicts the difference between a model’s Akaike’s Information Criterion (AIC) and that of the best-fitting model. (DOCX) [file pntd.0006098.s011.docx]

**S10 Table.** **Model selection for children infection with parasites.** Summary of the mixed effects model selection procedure for selecting the best parsimonious model for predicting parasite infection in the children population of Iguazú area. The Δ column depicts the difference between a model’s Akaike’s Information Criterion (AIC) and that of the best-fitting model.

| **Model** | **Variable groups** | **Fixed variables** | **logLink** | **AICc** | Δ **AICc** |
| --- | --- | --- | --- | --- | --- |
| M08 | Individual + Nutritional conditions + WASH + Exposure + Habits + House + Family + WASH | Age group + Obese or overweight + Tap water + Previous deworming treatment + Unsatisfied Basic Needs (UBN) + Single mother + Mother literacy + Overcrowding | -551.6 | 1127.8 | 0.0 |
| M07 | Child + Nutritional conditions + House + Habits + Exposure + House + Family + WASH + Environmental risk (PHCC level) | Age group + Obese or overweight + Unsatisfied Basic Needs (UBN) + Playing with soil + Previous deworming treatment + Overcrowding+ Tap water + Single mother + Co-contamination | -550.2 | 1129.2 | 1.4 |
| M06 | Child + Nutritional conditions + House + Habits + Exposure + House + Family + WASH | Age group + Obese or overweight + Unsatisfied Basic Needs (UBN) + Playing with soil + Previous deworming treatment + Overcrowding+ Tap water +Single mother + Mother literacy + Safe excreta disposal + Safe waste disposal | -551.1 | 1131.0 | 3.2 |
| M04 | Child + Nutritional conditions + House + Habits + Exposure | Age group + Obese or overweight + Unsatisfied Basic Needs (UBN) + Playing with soil + Previous deworming treatment | -556.7 | 1131.8 | 3.9 |
| M03 | Child + Nutritional conditions + House + Habits | Age group + Obese or overweight + Unsatisfied Basic Needs (UBN) + Playing with soil | -557.9 | 1132.1 | 4.2 |
| M05 | Child + Nutritional conditions + House + Habits + Exposure + House | Age group + Obese or overweight + Unsatisfied Basic Needs (UBN) + Playing with soil + Previous deworming treatment + Overcrowding | -559.4 | 1132.9 | 5.1 |
| M01 | Individual | Age group | -575.1 | 1160.3 | 32.4 |
| M02 | Child + Nutritional conditions | Age group + Obese or overweight | -581.1 | 1170.3 | 42.5 |
| M00 | Null model | 1 | -592.3 | 1188.5 | 60.7 |
