# Supplementary material for: Environmental and socio-demographic individual, family and neighborhood factors associated with children intestinal parasitoses at Iguazú, in the subtropical northern border of Argentina
Source: PLoS Negl Trop Dis. 2017 Nov 20;11(11):e0006098. doi: 10.1371/journal.pntd.0006098 (PMC5714390; doi:10.1371/journal.pntd.0006098)
Supplement: S11 Table — Summary of the mixed effects model selection procedure for selecting the best parsimonious model for predicting co-infection level in the children population of Iguazú area. The Δ column depicts the difference between a model’s Akaike’s Information Criterion (AIC) and that of the best-fitting model. (DOCX) [file pntd.0006098.s012.docx]

**S11 Table.** **Model selection for predicting children multi-parasitoses.** Summary of the mixed effects model selection procedure for selecting the best parsimonious model for predicting co-infection level in the children population of Iguazú area. The Δ column depicts the difference between a model’s Akaike’s Information Criterion (AIC) and that of the best-fitting model.

| **Model** | **Variable groups** | **Fixed variables** | **logLink** | **AICc** | Δ **AICc** |
| --- | --- | --- | --- | --- | --- |
| M08 | Child + Habits + WASH + Family + Environmental risk (PHCC level) | Age group + Playing with soil + Safe excreta disposal + Large family + Co-contamination | -521.7 | 1061.8 | 0.0 |
| M07 | Child + Habits + WASH + House + Family + Environmental risk (PHCC level) | Age group + Playing with soil + Safe excreta disposal + Unsatisfied Basic Needs + Large family + Co-contamination | -520.9 | 1062.2 | 0.4 |
| M06 | Child + Habits + WASH + House + Family | Age group + Playing with soil + Safe excreta disposal + Unsatisfied Basic Needs + Large family | -524.4 | 1065.1 | 3.3 |
| M04 | Child + Habits + WASH + House | Age group + Playing with soil + Safe excreta disposal + Unsatisfied Basic Needs | -529.9 | 1074.1 | 12.2 |
| M03 | Child + Habits + WASH | Age group + Playing with soil + Safe excreta disposal | -531.1 | 1074.3 | 12.5 |
| M05 | Child + Habits + WASH + House + Exposure | Age group + Playing with soil + Safe excreta disposal + Unsatisfied Basic Needs + Previous deworming treatment | -529.9 | 1076.1 | 14.2 |
| M01 | Individual | Age group | -536.2 | 1080.5 | 18.7 |
| M02 | Child + Nutritional conditions | Age group + Obese or overweight | -535.3 | 1080.7 | 18.9 |
| M00 | Null model | 1 | -545.2 | 1094.4 | 32.6 |
